# Genomic epidemiology of vancomycin-resistant *Enterococcus faecium* in Eastern Denmark from 2020 to 2022, and identification of *vanB* Tn*1549* insertion sites

**DOI:** 10.1007/s10096-025-05091-y

**Published:** 2025-04-01

**Authors:** Maja Johanne Søndergaard Knudsen, Jose Alfredo Samaniego Castruita, Ingrid Maria Cecilia Rubin, Sarah Mollerup, Helle Krogh Johansen, Rasmus L. Marvig, Karen Leth Nielsen, Barbara Juliane Holzknecht, Morten Hoppe, Michael Kemp, Henrik Westh, Mette Pinholt

**Affiliations:** 1 Department of Clinical Microbiology, Copenhagen University Hospital – Amager and Hvidovre, Copenhagen, Denmark; 2 Department of Virus & Microbiological Special Diagnostics, Statens Serum Institut, Copenhagen, Denmark; 3 Department of Genomic Medicine, Copenhagen University Hospital – Rigshospitalet, Copenhagen, Denmark; 4 Department of Clinical Microbiology, Copenhagen University Hospital – Rigshospitalet, Copenhagen, Denmark; 5 Department of Clinical Microbiology, Copenhagen University Hospital – Herlev and Gentofte, Copenhagen, Denmark; 6 Regional Department of Clinical Microbiology, Zealand University Hospital - Koege, Copenhagen, Denmark; 7 Department of Clinical Medicine, Faculty of Health and Medical Sciences, University of Copenhagen, Copenhagen, Denmark

**Keywords:** Vancomycin-resistant Enterococci, vancomycin-resistant *Enterococcus faecium*, VRE, *vanB*, Tn*1549*, Transposon insertion sites

## Abstract

**Background:**

We aimed to describe the genomic epidemiology of vancomycin-resistant *Enterococcus faecium* (VREfm) in Eastern Denmark from 2020 to 2022, identify and characterise the *vanB* Transposon *1549* (Tn*1549*) insertion sites among *vanB* VREfm clones and identify emerging VREfm clones.

**Methods:**

We analysed all VREfm from our routine diagnostic sequencing during the study period. Using the Seqsphere + v.8.2.0 software (Ridom GmbH, Münster, Germany, (http://www.ridom.de/seqsphere), minimum spanning trees were created to visualise clusters. Tn*1549* insertion sites were determined by in silico PCR. Nanopore sequencing was performed to assemble the regions surrounding Tn*1549*, which helped determine the insertion site locations.

**Results:**

We included 2,437 isolates in the study. A total of 463 isolates carried *vanA*, 1,963 isolates carried *vanB*, and 11 isolates carried both genes. Of all isolates carrying *vanB*, 254 isolates had the Tn*1549* inserted in the *araA2* gene, 1,604 in the *sir2* gene, and 116 in neither the *araA2* nor *sir2* genes. We identified eight chromosomal insertion sites other than in the *araA2* and *sir2* genes. Three isolates carried the Tn*1549* on plasmids. No emerging clones were found.

**Results:**

We have described the genomic epidemiology during the study period and identified ten chromosomal Tn*1549* insertion sites.

**Supplementary Information:**

The online version contains supplementary material available at 10.1007/s10096-025-05091-y.

## Introduction

*Enterococcus faecium* can cause nosocomial infections associated with intravenous catheter, invasive infections in the abdomen, and bacteraemia [1]. A Danish cohort study investigated enterococcal bacteraemia and found the incidence of *E. faecium* bacteraemia to be 6.6/100,000 person-years, and the 30-day mortality in this population to be 34.6% [2]. In another Danish study, the authors founds no difference in the 30-day mortality between patients with vancomycin-resistant *E. faecium* (VREfm) bacteraemia, and patients with vancomycin-susceptible *E. faecium* bacteraemia (37.6% vs.37.0%) [1]. Te in-hospital mortality rate of VREfm infections is 18%, risin to above 69% at 90 ays [3–5]. 

Vancomycin-resistance in *E. faecium* from human isolates is mainly caused by the acquisition of *vanA* or *vanB*. Studies have shown that the van operons can be transmitted between *E. faecium* strains by horizontal gene transfer [6, 7]. In this study, we focus on the *vanA* gene, which is located on Transposon *1546* (Tn*1546*) and most often carried on plasmids, and the *vanB* gene, which is located on Transposon *1549* (Tn*1549*), inserted in the *E. faecium* chromosome, or rarely on plasmids [8, 9]. 

From 2013, Denmark observed an increase in vancomycin-resistant *E. faecium* (VREfm) due to the emergence of several *vanA* VREfm clones. From 2019, *vanB* VREfm started to increase and outcompeted *vanA* VREfm [10, 11]. 

The emergence of *vanB* VREfm has challenged rapid diagnostic of VREfm from rectal screening samples by PCR since *vanB* is present in multiple gut commensals other than *E. faecium*. As previously described, a multiplex PCR, targeting both *vanB*, and the specific Tn*1549* insertion sites in the chromosome in the two most dominating *vanB* clones (a L-arabinose isomerase gene (*araA2*), and a silent information regulator gene (*sir2*) in our area, was developed at the DCM Hvidovre Hospital to overcome this diagnostic challenge [8, 12]. 

Accurate and rapid diagnostic of VREfm performed directly on rectal swabs (within 12 h) is crucial to maintain infectious hygiene precautions in a hospital setting, and to prevent hospital outbreaks [13]. In our regions, we carry out contact isolation precautions for all patients that are carriers of or infected with VREfm. In one of the regions’ four DCMs (Hvidovre Hospital) we use the results from the multiplex PCR to determine which VREfm clone patients are colonised or infected with. This allows for timely analysis of VREfm dissemination in hospital wards. In addition, when we need to perform cohort isolation in case of lack of single rooms in the wards, we use the results from the multiplex PCR. In those cases, we perform cohort isolation of patients that have the same *van* gene and Tn*1549* insertion site on the multiplex PCR. We use VREfm whole genome sequencing (WGS) for continuous surveillance of VRE on a regional level. This is important to identify the VREfm clone-distribution in our hospitals and emergence of new clones that require diagnostic changes.

The aims of this study were to describe the genomic epidemiology of VREfm in Eastern Denmark, from 2020 to 2022 and to identify emergence of new clones, especially *vanB* clones with non-*araA2*/non-*sir2* Tn*1549* insertion sites. Furthermore, we aimed to identify and characterise the Tn*1549* insertion sites among *vanB* VREfm isolates in our collection to evaluate how many different Tn*1549* insertion sites that are present in our *vanB* VREfm collection and to determine the proportion of *vanB* VREfm that will be identified by the laboratory developed multiplex-PCR.

## Methods

### Setting and collection of data

In Eastern Denmark we perform routine diagnostic sequencing of one VREfm isolate per patient per year. In the cases, where patients have samples positive for both *vanA* and *vanB* genes within the same year, we sequence both isolates in line with our active surveillance strategy. The routine sequencing applies to both screening and clinical isolates. Infection prevention and control measures include isolation of VREfm positive patients and screening of patients from the same room, whenever VREfm is found in a sample from a patient that was not isolated. If a patient from the same room has a positive sample for VREfm, we screen all patients on the ward. In periods with VREfm outbreaks, we perform weekly screenings. We screen patients at admission if they have had a VREfm positive sample within the past six months. In this retrospective study we collected all sequences from VREfm isolates from January 2020 until June 30, 2022, from Eastern Denmark (Region of Zealand and the Capital Region of Denmark with 2.7 million inhabitants, approximately 50% of the Danish population). We have four departments of clinical microbiology (DCMs), all of which have collected isolates that are represented in the study, and the DCMs serve a total of 15 hospitals as well as all general practitioners in the area.

### Whole genome sequencing and bioinformatical methods

We used the Nextera XT DNA Sample Preparation Kit (Illumina, San Diego, California, U.S.A) to prepare libraries, and the isolates were sequenced either on the Illumina MiSeq or the NextSeq platform (Illumina Inc., San Diego, USA) in paired end reads. The sequences were assembled with SKESA v.2.2 or SPADES depending on the DCM [14, 15]. We used quality control parameters, and we included only isolates with N50 above 10,000, and genome sizes between 2.7 and 3.3 million base pairs. We used the Seqsphere + v.8.2.0 software (Ridom GmbH, Munster, Germany, http://www.ridom.de/seqsphere/) to analyse all isolates based on the formerly published scheme for *E. faecium* consisting of 1,423 core genome Multi Locus Sequence Type (cgMLST) target genes [16, 17]. We created Minimum Spanning Trees (MST) to visualise cgMLST CT clusters with the setting of maximum 20 alleles between isolates. Genome annotation was performed in the assemblies using Prokka v.1.14.5, and the annotating proteins in Aus0085 [18, 19]. Regions surrounding Tn*1549* were blasted using ncbi-blast v.2.8.1 [20]. Insertions sites were visualised using BRIG v.0.95, R v.4.3.0 and gggenes library [21–23]. 

### Analysis of the Tn*1549* insertion sites

We used the multiplex PCR primer sets to perform in silico PCR on all *vanB* isolates to determine which isolates belonged to the dominating Tn*1549* insertion sites in *araA2* or *sir2* genes in the chromosome [8, 12]. Subsequently, we analysed the non-*araA2*/non-*sir2* isolates. The regions surrounding Tn*1549* were used to predict the inserted sites in these isolates. Based on the MST, we proceeded with nanopore sequencing on 12 representative isolates without the Tn*1549* insertion site in *araA2* or *sir2* to construct a better assembly of the genome and the insertion sites (Table 1).

### Nanopore sequencing

Genomic DNA libraries were built using the Rapid Barcoding Kit and sequenced using a MinION (Oxford Nanopore Technology, Oxford, UK) with a R9.4.1 flow cell. Hybrid assemblies were done using the Illumina and Nanopore reads with Unicycler [24]. Mauve was used to compare the hybrid assemblies and inspect genome rearrangements [25]. Visualisation of the genomes was done by BLAST Ring Image Generator (BRIG) [26]. 

## Results

### Genomic epidemiology of *VanA* and *VanB*

**Fig. 1:**
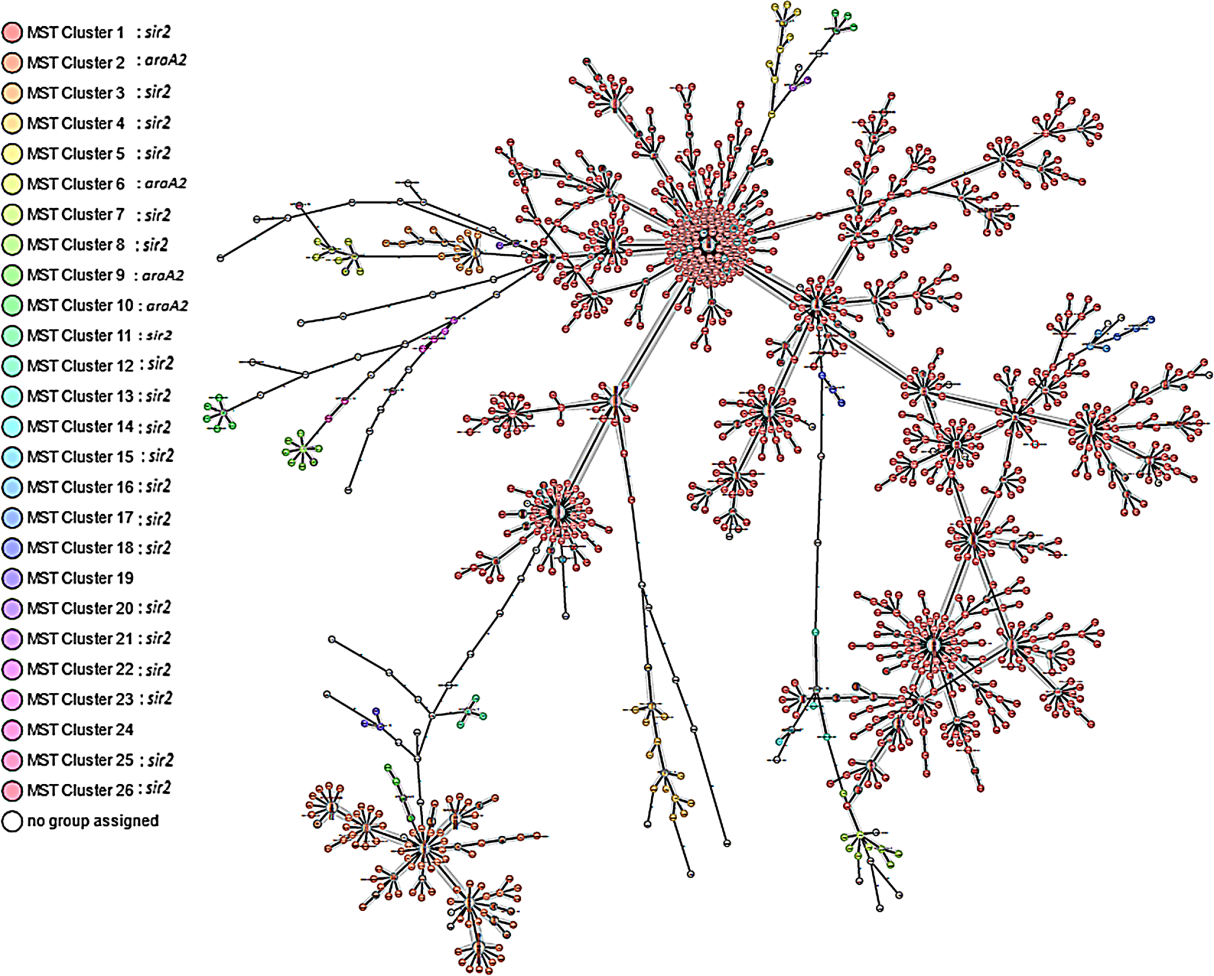
Shows the minimum spanning tree of the *vanB* and *vanAB* isolates. The isolates are coloured according to the clustering of the isolates by the core genome multilocus sequence types. The vast majority of the cluster 1 isolates has the Tn*1549* inserted in *sir2*, and the vast majority of the isolates in cluster 2 has the Tn*1549* inserted in *araA2*

In total 2,437 VREfm isolates were included in the study. *vanB* VREfm was dominant with 1,963 (81%) isolates; however, *vanA* was still present with 463 isolates (19%). Eleven isolates carried both *vanA* and *vanB*. Most of the isolates in the *vanB* and *vanAB* subgroup belonged to ST80/CT2406 (*n* = 1,614, 82%/*n* = 1,180, 60%). The MST of *vanB* and *vanAB* isolates is presented in Fig. 1, where cluster 1 is mainly represented with ST80 isolates.

The most prevalent Sequence Types (ST) and cgMLST Complex Types (CT) in *vanA* and *vanAB* carrying isolates were the ST1421/CT1134 (*n* = 307, 65%/*n* = 305, 64%). The dominating ST1421 clone was the vancomycin-variable clone that was introduced to our area in the 2016 [11, 27]. The *vanA* carrying isolates are presented in the supplementary figure, where most of the ST1421/CT1134 are found in cluster 1. The 11 isolates carrying both *vanA* and *vanB* was a diversified group that belonged to four different ST, and five different CT.

### Tn*1549* insertion site

Of the 1,974 *vanB* isolates, 254 (13%) isolates had the Tn*1549* inserted in *araA2*, and 1,604 (81%) isolates had the Tn*1549* inserted in the *sir2*. Thus, the multiplex PCR targeting *vanB* and the *araA2*/*sir2* insertion sites identifies 94% of the *vanB* VREfm isolates in VREfm from Eastern Denmark. We found the *sir2* insertion sites in 20 different MST clusters including the biggest cluster 1. The *araA2* insertion site was found in four different MST clusters including the cluster 2 (Fig. 1).

**Fig. 2:**
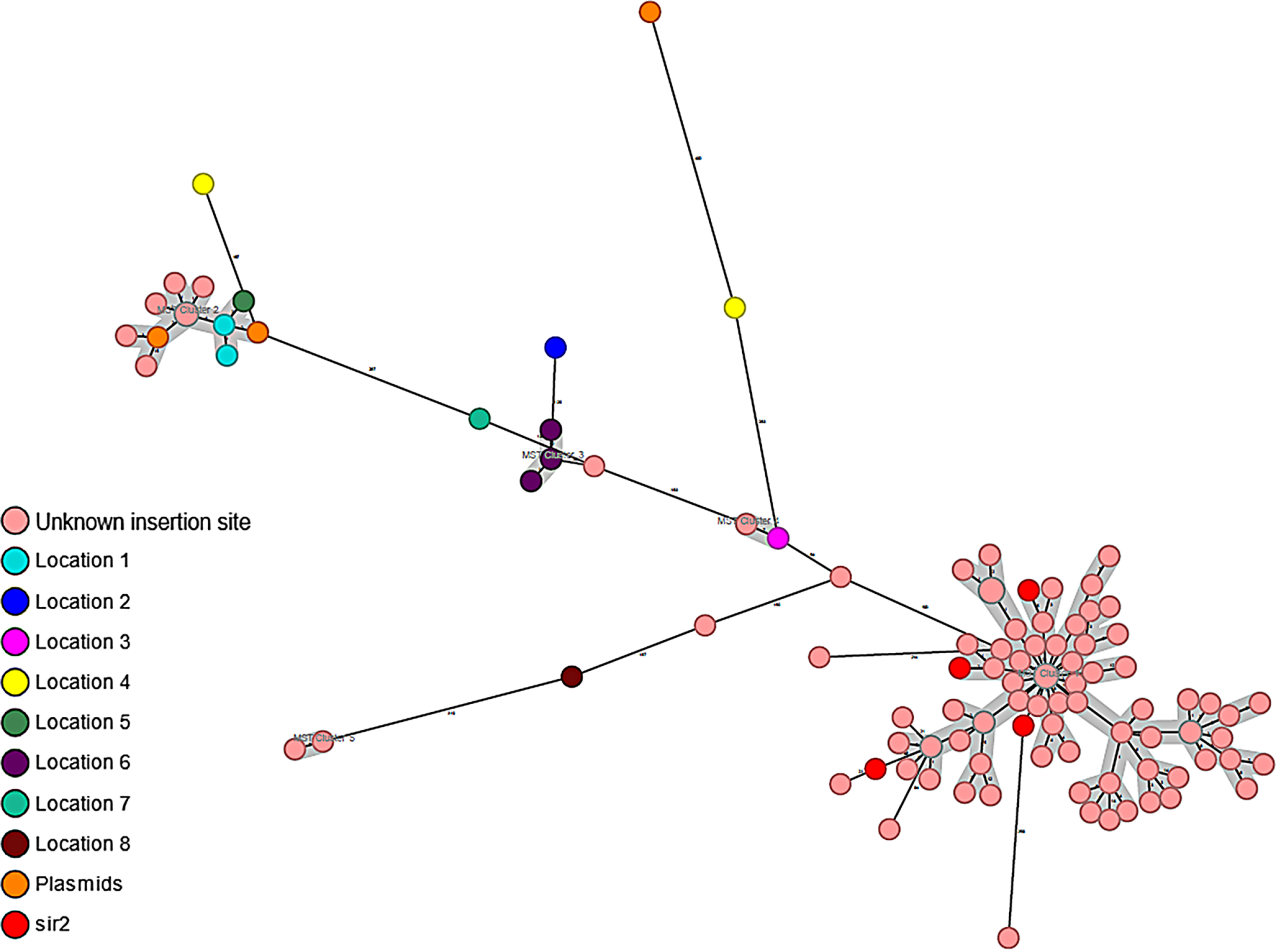
Shows the minimum spanning tree of the non-*araA2*/non-*sir2* isolates. The isolates are coloured according to the Tn*1549* insertion sites determined by analysis of contigs assembled from Illumina data (7 isolates), or hybrid assembly with Illumina and Nanopore data (12 isolates). The isolates with Tn*1549* insertion site in *sir2* (red) are all located in the biggest MST cluster

In total, we failed to find a complete in silico PCR product in 116 samples, either because they had a non-*araA2*/non-*sir2* insertion site, or incomplete PCR products were present on the contigs (Fig. 2). In these samples, ST80/CT2406 (*n* = 84, 72.4%/*n* = 64, 55.1%) were most frequent, as was the case for the *vanB* isolates with the *sir2* insertion site. None of these isolates had undergone the routine analysis with the multiplex PCR detecting both *araA2* and *sir2* insertion sites.

### VREfm clones with non-*araA2*/non-*sir2* Tn*1549* insertion sites

**Fig. 3:**
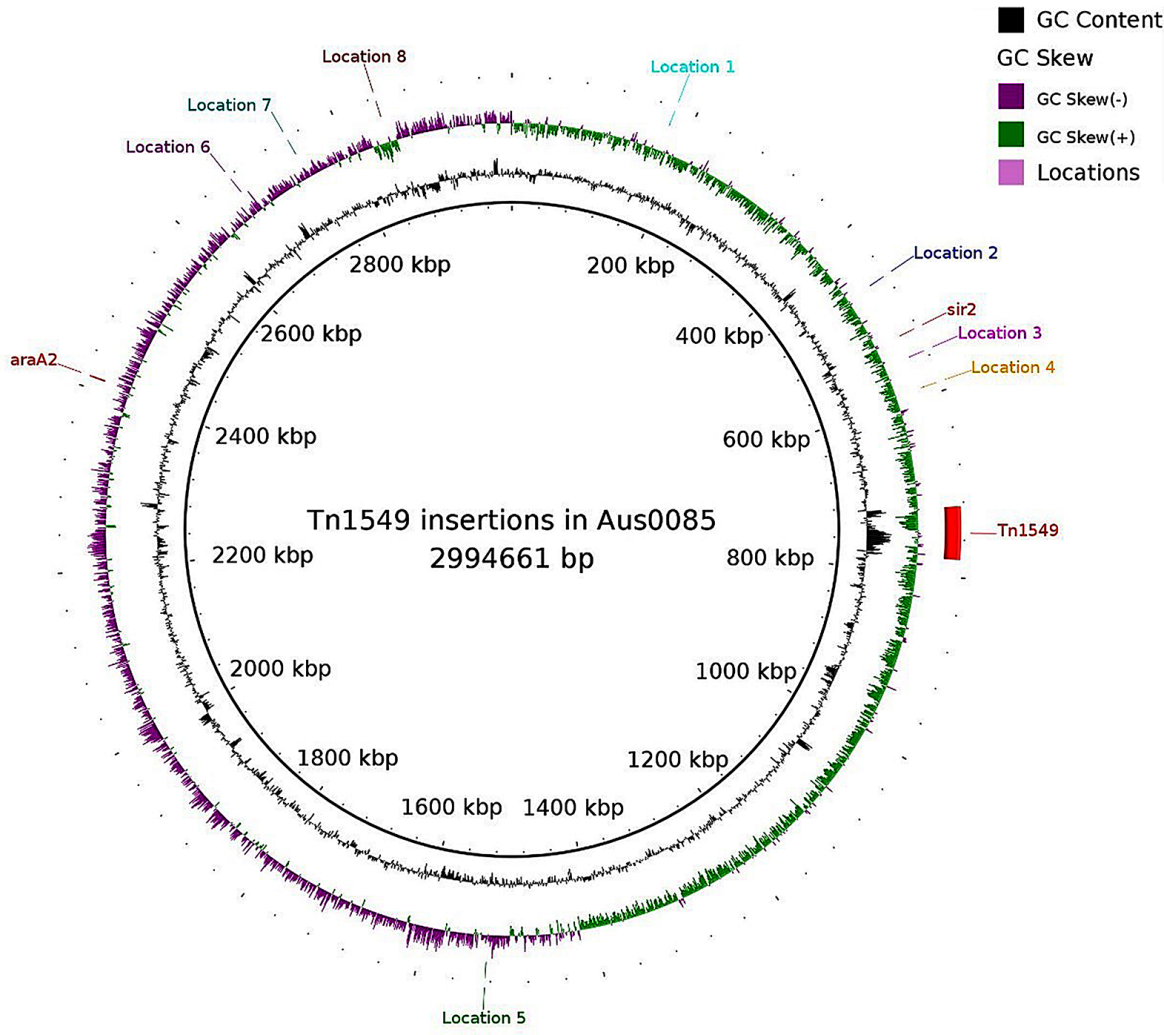
Shows the location of the *araA2* Tn*1549* insertion site, the *sir2* insertion site, and the eight other chromosomal insertion sites that we have identified, based on the Aus0085 assembly (NC_021994)

We were able to identify the Tn*1549* insertion sites in 19 of the 116 non-*araA2*/non-*sir2* isolates using Nanopore and Illumina sequencing (12 with Nanopore, and 7 with Illumina). There were eight different predicted locations where Tn*1549* was inserted in the genome. In three isolates the transposon was predicted to be located on a plasmid. All non-*araA2*/non-*sir2* isolates are presented in the MST in Fig. 2, where the isolates are coloured according to the insertion site. The location of the insertion sites identified in 19 isolates is shown on Fig. 3 and in Table 1. In the Table 1, we have marked the isolates that have undergone Nanopore sequencing. Four isolates turned out to have the insertion site in *sir2*, although the in silico PCR failed to find a complete PCR product (coloured red in Fig. 2). Three of these isolates had been Nanopore sequenced.

**Table 1:** Overview of the Tn*1549* insertion sites based on the Aus0085 assembly, as well as strand (+/-), genes or loci, where Tn*1549* has been inserted, the ST, and the CT for each isolate. ^a^Tn*1549* is located next to the *sir2* gene. ^b^Tn*1549* is located next to a genomic rearrangement. ^c^Nanopore and illumina reads were used for the assembly

Sample	Tn1549 insertion site	Strand	Locus or gene	MLST ST	cgMLST CT
V3969^c^	177,141 (location 1)	-	EFAU085_RS00880/EFAU085_RS00885	1421	1134
VRE5237	177,141 (location 1)	-	EFAU085_RS00880/EFAU085_RS00885	1421	1134
V4484^c^	467,030 (location 2)	-	EFAU085_RS02205	117	991
VE2908	528,269^a^ (sir2)	+	*sir2*	Unknown	5203
VRE5912^c^	528,269^a^ (sir2)	+	*sir2*	80	2406
VE2293^c^	528,269^a^ (sir2)	+	*sir2*	80	4836
VE2295^c^	2,446,246^a, b^ (sir2)	+	*sir2*	80	2946
VE2445	554,193 (location 3)	-	EFAU085_RS02565/EFAU085_RS02575	897	1860
V3997^c^	589,859 (location 4)	-	EFAU085_RS02740/EFAU085_RS02745	17	3115
VRE6733	589,859 (location 4)	-	EFAU085_RS02740/EFAU085_RS02745	612	2362
VE2418^c^	1,572,492 (location 5)	-	EFAU085_RS07530/EFAU085_RS07820	1421	1134
V3832	2,672,254 (location 6)	+	EFAU085_RS13235/EFAU085_RS18160	117	1180
V3870^c^	2,672,254 (location 6)	+	EFAU085_RS13235/EFAU085_RS18160	117	1180
VRE5216	2,672,254 (location 6)	+	EFAU085_RS13235/EFAU085_RS18160	117	1180
VRE7066^c^	2,745,504 (location 7)	-	EFAU085_RS13595	117	5149
RHVRE034^c^	2,868,125 (location 8)	-	*fss3*/EFAU085_RS17090	1731	Unknown
VE2234	125,278 (plasmid 1)	-	EFAU085_RS15400	1421	1134
VRE5694^c^	25,484 (plasmid 3)	-	EFAU085_RS15970	1421	1134
VRE6980^c^	4705 (plasmid 1)	+	EFAU085_RS14720/EFAU085_RS14730	1693	2532

Using the genome annotations, we could see two variants of the Tn*1549* sequence from samples that failed to find a complete *araA2* or *sir2 in silico* PCR product. One of the variants has an ISL3 family transposase inserted in the opposite strand between *vanS* and *vanY*. Most samples with the predicted variant with the ISL3 family transposase belong to ST80 (95.8%).

## Discussion

In this study we have investigated the genomic epidemiology of VREfm in two regions in the Eastern part of Denmark from 2020 to 2022. We have characterised the Tn*1549* insertion sites among the *vanB* clones and evaluated the coverage of our current multiplex PCR. The majority of the VREfm isolates carried *vanB* and had the Tn*1549* inserted in *sir2*. Our multiplex PCR correctly identified 94.1% of the *vanB* isolates. We have identified eight chromosomal insertion sites other than *araA2* and *sir2*. None of these isolates are currently a part of an emerging clone, and there is no evidence suggesting these isolates have an increased fitness in comparison to the isolates with *araA2* and *sir2* insertion sites in our collection of isolates from 2020 to 2022.

Interestingly, in the subgroup of non-*araA2*/non-*sir2* isolates we discovered some isolates that turned out to have the insertion site in *sir2* after reassembling the genomes using Nanopore technology (coloured red on the Fig. 2). These isolates were found in the largest MST cluster of the non-*araA2*/non-*sir2* subgroup. We hypothesize that the isolates in this MST cluster have Tn*1549* inserted in the *sir2* gene, but they show a negative result in the in silico PCR due to the PCR product being located in two different contigs in their assemblies. Like the *sir2* clone, most of the isolates in this MST cluster belonged to ST80/CT2406 [12]. We would hypothesize that these isolates would turn positive in the multiplex PCR, which thereby could identify more than 94% o the *vanB* isolates in Eastern Denmark.

International colleagues from Germany, the Netherlands, and Australia have also identified the Tn*1549* insertion sites in their *vanB* isolates [28–30]. Two of our isolates have the Tn*1549* inserted between gene lacI (GenBank locus tag: EFAU085_RS02740) and a gene encoding a hypothetical protein (GenBank locus tag: EFAU085_RS02740), which is also one of the insertion sites that Zhou et al. and Bender et al. found in their studies [29, 30]. We also found that isolate VRE5694 had the Tn*1549* inserted in a plasmid gene that encodes for a recombinase family protein (GenBank locus tag: EFAU085_RS15970) or DNA invertase Pin gene in Zhou et al. [29]

Our study is limited by the geographical area, as we only have isolates from Eastern Denmark. In the future we wish to apply this method to a larger collection of isolates from different countries to learn more about preferred insertion sites for Tn*1549* in the *E. faecium* chromosome. Since we found ten different insertion sites in our collection, we hypothesise that we might find an increased number of insertion sites in isolates from a much larger geographical area. Another limitation is that we did not have the possibility to perform Nanopore sequencing on all 116 non-*araA2*/non-*sir2* isolates, therefore we had to select a subgroup from different MST clusters. The strength of our study is that we include isolates from all DCMs in Eastern Denmark serving approximately half of the Danish population. Our results show that most of the VREfm isolates belong to the two biggest clones, allowing us to perform VREfm diagnostics with our multiplex PCR. However, the present study showed that multiple clusters have the same Tn*1549* insertion sites (*araA2* or *sir2*), thus, the multiplex PCR cannot be used as a precise typing tool. The fact that we find the *sir2* insertion site in 20 different MST clusters, and the *araA2* insertion site in four different clusters, indicate that either the Tn*1549* has a few preferred insertion sites in the *E. faecium* chromosome, or the Tn*1549*/*sir2* has spread to vancomycin-susceptible *E. faecium* through recombination events.

In our collection of isolates from 2020 to 2022 we found no emerging *vanB* VREfm clones that would pose a threat to the accuracy of our multiplex PCR. With the results from this study and the identification of eight insertion sites other than *araA2* and *sir2*, we have contributed to the knowledge of possible insertion sites for the *vanB* Tn*1549*. We will continue the surveillance of the genomic epidemiology of VREfm, as well as the accuracy of our multiplex PCR. In case of a decrease in the accuracy and if a new dominating clone emerges, we will be able to update our multiplex PCR, to continue to provide rapid VREfm results to our infection control organisations.

## Conclusion

In the study period, 94% of *vanB* isolates had the Tn*1549* inserted in either *araA2* or *sir2* and were identified by our multiplex PCR. We identified eight insertion sites other than *araA2* and *sir2* and found no emerging clones within these insertion sites. We will continue the surveillance, and update our multiplex PCR, when and if this changes.

## Electronic supplementary material

Below is the link to the electronic supplementary material.

Supplementary Material 1

Supplementary Material 2

## Data Availability

The sequences of the included isolates have been uploaded to NCBI Bioprojects PRJNA1023182, PRJNA1046682, PRJNA1050871, PRJNA1225185, PRJNA686881, PRJNA775877, and PRJEB63096. For details on specific isolates, please contact the corresponding author.
