# Supplementary material for: Genomic epidemiology of vancomycin-resistant Enterococcus faecium in Eastern Denmark from 2020 to 2022, and identification of vanB Tn1549 insertion sites
Source: Eur J Clin Microbiol Infect Dis. 2025 Apr 1;44(6):1425–32. doi: 10.1007/s10096-025-05091-y (PMC12116600; doi:10.1007/s10096-025-05091-y)
Supplement: Supplementary file 2 — Supplementary Material 2 [file 10096_2025_5091_MOESM2_ESM.docx]

**Fig. S1** shows the minimum spanning tree of the *vanA* isolates. The isolates are coloured according to the clustering of the isolates by the core genome multilocus sequence types.
